# Change in Children’s Self-Concept, Body-Esteem, and Eating Attitudes Before and 4 Years After Maternal RYGB

**DOI:** 10.1007/s11695-018-3348-z

**Published:** 2018-06-17

**Authors:** Fanny Sellberg, Ata Ghaderi, Mikaela Willmer, Per Tynelius, Daniel Berglind

**Affiliations:** 1 Department of Public Health Sciences, Karolinska Institutet, K9, Social Medicine, 171 77 Stockholm, Sweden; 2 Department of Public Health Sciences, Karolinska Institutet, Solnavägen 1E, 113 65 Stockholm, Sweden; 3 Department of Clinical Neuroscience, Karolinska Institutet, 171 77 Stockholm, Sweden; 4 Department of Health and Caring Sciences, University of Gävle, 801 76 Gävle, Sweden; 5 Centre for Epidemiology and Community Medicine, Stockholm County Council, Box 45436, 104 31 Stockholm, Sweden

**Keywords:** Bariatric surgery, Roux-en-Y gastric bypass, Children, Longitudinal, Psychosocial functioning, Eating behavior

## Abstract

**Introduction:**

The aim of the present study was to look at longitudinal changes in children’s self-concept, body-esteem, and eating attitudes before and 4 years after maternal RYGB surgery.

**Methods:**

Sixty-nine women and 81 appurtenant children were recruited from RYGB waiting lists at 5 hospitals in Sweden. Families were visited at home pre-surgery, 9 months, and 4 years post-maternal RYGB to measure BMI. Furthermore, all participating family members completed questionnaires. Mothers’ questionnaires measured eating behavior, depression, anxiety, and sleep quality, and children’s questionnaires measured body-esteem, self-concept, and eating attitudes.

**Results:**

Thirty-five/sixty-nine mothers and 43/81 children participated in all 3 measurements. Mothers reduced their BMI from pre-surgery (39.2) to 9 months (27.0) and 4 years post-surgery (27.4). Children’s prevalence of overweight/obesity was lower 9 months post-surgery (48.8%) but at the same levels again 4 years post-surgery (58.1%), compared to pre-surgery (58.1%). The same rebound pattern was seen among children’s eating attitudes, mothers’ symptoms of depression and anxiety, and sleep quality. We found no correlations between mothers’ BMI or eating behavior and children’s BMI or eating behavior.

**Conclusion:**

Children’s prevalence of overweight/obesity and eating attitudes improves soon after their mothers’ RYGB, but then return to pre-surgery levels at 4 years post-surgery, as do mothers’ sleep quality and symptoms of depression and anxiety, even though their weight loss was maintained.

**Electronic supplementary material:**

The online version of this article (10.1007/s11695-018-3348-z) contains supplementary material, which is available to authorized users.

## Introduction

Bariatric surgery has become an increasingly common treatment method for severe obesity. Roux-en-Y gastric bypass (RYGB) is an established and frequently used method and accounted for over 64% of all bariatric procedures in Sweden 2016 [1]. The vast majority (75%) of all bariatric surgery in Sweden is performed on women [2].

With refinements of surgical techniques and reductions in surgical complications [1], the focus of research is increasingly turning towards the psychosocial aspects of bariatric surgery. It has previously been shown that patients’ depressive symptoms are reduced after surgery and that health-related quality of life is improved also over longer periods, although most effect is seen during the first year post-surgery [3]. However, less is known about how children might be affected by a family member’s surgery and whether any such effects are maintained long-term.

Woodard et al. investigated family members’ weight change after a parent’s RYGB surgery and found that children had a lower BMI than expected 1 year after their parent’s RYGB. However, this effect did not reach statistical significance. They also found that children more frequently reported currently being on a diet after parental surgery but found no change in quality of life [4]. Willmer et al. investigated children of mothers who underwent RYGB and found that they had a lower relative risk of overweight and obesity 9 months post-surgery [5]. They also found an improvement in body-esteem and eating attitudes among boys, but not among girls, 9 months after maternal surgery [5, 6]. Since children’s levels of obesity and overweight and eating behavior seem to be associated with their parents’ BMI [7] and eating behavior [8] at short-term follow-ups, it is of interest to investigate if changes in mothers’ eating behavior and weight loss after RYGB, at long-term follow-ups, would be associated with their children’s weight status and well-being. If so, maybe maternal bariatric surgery could additionally act as prevention for their children’s obesity.

To summarize, few studies have investigated the familial effects of bariatric surgery, and no study that we are aware of has followed families for more than 1 year. It is of importance to study surgical effects over longer periods since many problems, for example eating behavior [9, 10] and weight regain [11], commonly arise after a year or two.

Thus, the first aim of the present study was to study the changes in children’s eating attitudes, self-concept, body-esteem, and weight status before, 9 months, and 4 years after maternal RYGB surgery. The second aim was the investigation of mothers’ eating behavior, sleep quality, changes in relationship status, and symptoms of depression and anxiety over the same time period. Finally, the third and last aim was to investigate if BMI and eating behavior among mothers correlate with children’s BMI or eating behavior.

## Materials and Methods

This observational study recruited 69 women with their families from waiting lists for RYGB surgery at 5 different hospitals in Sweden between April 2011 and October 2012. Inclusion criteria included being eligible for primary RYGB, being able to speak and understand Swedish, and having at least one child aged 7–14 years at the time of recruitment. If the women were living with a partner, their partners were also asked to participate. The study was approved by the Stockholm Regional Ethical Review Board (no 2009/1472-31/3) and all participants gave written informed consent at the first and last data collection time point.

Weight, height, and waist circumference were measured objectively in the families’ homes by 2 researchers at 3 months pre-surgery and 9 months and 4 years post-surgery (calibrated scale: VB2-200-EC, Vetek AB, Väddö, Sweden; portable stadiometer: Seca 213, Seca, Chino, CA, USA). All participants were asked to fill in questionnaires and either return them by mail or give them to the researchers at the home visit. All participants who completed baseline data were invited for the 9-month and 4-year follow-up, with the exception of partners who divorced or separated from the operated mothers before the follow-up.

### Questionnaires

The women completed the Three-Factor Eating Questionnaire (TFEQ) [12] measuring cognitive restraint (CR), emotional eating (EE), and uncontrolled eating (UE), the Hospital Anxiety and Depression Scale (HADS) [13, 14], and the Karolinska Sleep Questionnaire (KSQ) [15].

Children completed the Swedish version of the Body Esteem Scale (BES) [16, 17], including 23 items and measuring weight concerns (BES-W), appearance (BES-APP), and attribution (BES-ATT). BES is a valid and reliable scale for male and female adolescents of a wide range of age [16]. They also completed the Beck Youth Inventory (BYI-S) [18] and the Children’s Eating Attitude Test (ChEAT) [19, 20]. ChEAT was developed to measure a broad range of disordered eating attitudes associated with anorexia nervosa and bulimia nervosa and have shown good internal reliability and good concurrent validity against questions of weight management behavior, in a population of middle school girls, mean age 13.2 years [21]. BYI-S was developed for self-reporting by children between the ages of 7 and 14. The scale has shown acceptable validity and reliability (median test–retest reliability coefficients exceeded the minimum criterion of 0.80) [22]. For the current study, we only used the self-concept subscale of the BYI.

### Statistical Analysis

BMI was calculated as weight (kg) divided by height squared (m^2^), and children’s obesity and overweight were calculated and defined according to age- and sex-specific cut-off points developed by Cole et al. [23]. A score of ≥ 8 on either of the HADS subscales for depression and anxiety was used as the cut-off for symptoms of depression or anxiety, respectively [14]. BYI-S scores are presented as total score and in percentile ranks, derived from a standard population of 2400 Swedish school children. A score between the 26th and the 89th percentiles was used as reference category [24]. All other questionnaires are presented as continuous sum scores, means, and standard deviations. Categorical variables (same partner as before surgery, anxious symptomatology, depressive symptomatology, overweight, and obesity) are presented as number and percentage. Differences between first (baseline) and second measurement, first and third measurement, and between second and third measurement were analyzed with paired *t* tests for continuous variables and with McNemar’s test for dichotomous variables. By design, the tests are controlled for fixed factors remaining constant (genetic and to some extent environmental factors) within subjects between the measurements. Individuals with missing data on a certain scale were excluded from analysis of results of that scale. Pearson correlation was used to assess relationships between the mothers’ BMI and the children’s BMI, as well as between mothers’ eating behavior and their children’s eating behavior. All statistical analyses were conducted using STATA 14.1 (StataCorp).

## Results

Thirty-five out of 69 women (mean age 39.5 years) and 43 out of 81 of their children (mean age 10.1 years, range 7.1–15.0 at recruitment) participated in all three measurement points.

### Mothers’ Changes in Weight, Eating Behavior, Sleep Quality, and Symptoms of Depression and Anxiety

Table 1 shows women’s and children’s characteristics and differences between the first and second measurements, the second and third measurement, and between the first and third measurement. The women had lost an average of 32.5 kg 4 years post-surgery and reduced their BMI correspondingly with 11.8 kg/m^2^. BMI remained relatively stable between 9 months and 4 years post-surgery in this group of women. Twenty-seven percent of the women showed symptoms of anxiety and 19% of depression 4 years post-surgery (see Table 2). This was not significantly lower than pre-surgery (*p* = 0.21 and *p* = 0.48, respectively), but significantly higher than 9 months post-surgery (*p* = 0.05 and *p* = 0.01). The women’s self-reported sleep quality improved post-surgery, with a larger effect seen after 9 months than after 4 years (from 76.6 points before surgery to 90.8 points and 85.9 points 9 months and 4 years post-surgery, respectively). The women also showed improvement of Emotional Eating (TFEQ-EE) (from 15.5 to 9.6 points) and Uncontrolled Eating (TFEQ-UE) (from 21.9 to 13.8 points). In contrast, cognitive Restraint (TFEQ-CR) (from 13.1 to 13.5 points) did not increase 4 years post-surgery compared to pre-surgery (Table 2).

**Table 1:** Anthropometric measurements among women and children 3 months before and 9 and 48 months after maternal Roux-en-Y Gastric Bypass surgery. Differences tested with pairwise *t* test and McNemar’s test

Variables	Participated/recruited	Pre-surgery, 1st	9 months post-surgery, 2nd	4 years post-surgery, 3rd	1st compared to 2nd	2nd compared to 3rd	1st compared to 3rd
Women							
BMI	35/69	39.2 (3.2)	27.0 (3.0)	27.4 (3.8)	− 12.2 (*p* < 0.001)	0.4 (*p* = 0.524)	− 11.8 (*p* < 0.001)
Weight	35/69	107.3 (13.0)	73.7 (9.8)	74.8 (12.2)	− 33.6 (*p* < 0.001)	1.1 (*p* = 0.473)	− 32.5 (*p* < 0.001)
Children							
BMI	43/81	20.3 (3.7)	20.6 (3.5)	23.5 (4.5)	0.6 (*p* = 0.187)	2.8 (*p* < 0.001)	3.2 (*p* < 0.001)
Overweight/obese	43/81	58.1% (25)	48.8% (21)	58.1% (25)	− 9.3% (*p* = 0.103)	9.3% (*p* = 0.206)	0% (*p* = 1.000)
Overweight	43/81	46.5% (20)	37.2% (16)	34.9% (15)	− 9.3% (*p* = 0.206)	− 2.3% (*p* = 0.796)	− 11.6% (*p* = 0.197)
Obese	43/81	11.6% (5)	11.6% (5)	23.3% (10)	0% (*p* = 1.000)	11.7% (*p* = 0.059)	11.7% (*p* = 0.059)

Data presented as means (standard deviation) or percentage (numbers)

**Table 2:** Women’s eating behavior, depression, anxiety, and sleep quality, 3 months before and 9 and 48 months after Roux-en-Y Gastric Bypass surgery. Differences within women tested with pairwise *t* test and McNemar’s test

Variables women	Participated/recruiter	Pre-surgery, 1st	9 months post-surgery, 2nd	4 years post-surgery, 3rd	1st compared to 2nd	2nd compared to 3rd	1st compared to 3rd
Same partner as pre-surgery	31/69		24 (77.4%)	21 (67.7%)			
HADS^a^ total score	29/69	12.2 (7.7)	5.9 (5.7)	9.2 (8.5)	− 6.3 (*p* < 0.001)	3.3 (*p* = 0.023)	− 3.0 (*p* = 0.092)
Anxiety score	30/69	7.1 (5.2)	4.0 (4.4)	5.6 (5.5)	− 3.1 (*p* = 0.004)	1.6 (*p* = 0.073)	− 1.5 (*p* = 0.187)
Depression score	32/69	5.1 (3.2)	1.7 (1.8)	3.5 (3.5)	− 3.4 (*p* < 0.001)	1.8 (*p* = 0.008)	− 1.6 (*p* = 0.030)
Anxious	30/69	12 (40%)	4 (13.3%)	8 (26.7%)	− 8 (*p* = 0.011)	4 (*p* = 0.046)	− 4 (*p* = 0.206)
Depressed	32/69	8 (25%)	0	6 (18.8%)	− 8 (*p* = 0.005)	6 (*p* = 0.014)	− 2 (*p* = 0.480)
Sleep score	33/69	76.6 (16.9)	90.8 (9.6)	85.9 (13.9)	14.3 (*p* < 0.001)	− 4.9 (*p* = 0.051)	9.3 (*p* = 0.003)
Eating behavior total score	33/69	100.3 (22.4)	70.5 (14.4)	74.0 (19.4)	− 29.7 (*p* < 0.001)	3.4 (*p* = 0.264)	− 26.3 (*p* < 0.001)
Uncontrolled eating score	30/69	21.9 (6.2)	12.4 (2.8)	13.8 (4.2)	− 9.5 (*p* < 0.001)	1.5 (*p* = 0.035)	− 8.3 (*p* < 0.001)
Cognitive restraint score	29/69	13.1 (2.5)	14.7 (3.8)	13.5 (3.4)	1.6 (*p* = 0.074)	− 1.2 (*p* = 0.079)	0.3 (*p* = 0.646)
Emotional eating score	33/69	15.5 (5.1)	8.8 (2.9)	9.6 (3.8)	− 6.7 (*p* < 0.001)	0.8 (*p* = 0.136)	− 5.8 (*p* < 0.001)

^a^The Hospital Anxiety and Depression Scale

Beck Depression Inventory-II. Data presented as means (standard deviation) or numbers (percentage)

Twenty-one women (67.7%) had the same partner 4 years post-surgery as they did before surgery (out of the participants who reported their relationship status, see Table 2).

### Children’s Changes in Self-Esteem, Self-Concept, Weight Status, and Eating Behavior

As for the mothers, children’s prevalence of overweight and obesity decreased 9 months post-surgery, and then increased again 4 years post-surgery, although these results failed to reach statistical significance (*p* = 0.103 and *p* = 0.206, respectively), see Table 1. More children were classified as obese 4 years after the mothers’ surgery, compared to both pre-surgery and 9 months post-surgery (*p* = 0.059). Table 3 shows children’s self-concept, body-esteem and eating attitudes, and differences between the three measurement points. Children’s self-concept and body-esteem (all subcategories, weight, attribution, and appearance) decreased by time after the mothers’ surgery. Eating behavior first improved 9 months post-surgery, but then deteriorated again 4 years post-surgery. Thus, following the same rebound pattern as mothers’ eating behavior (UE and EE) although children’s eating behavior was even poorer after 4 years compared with pre-surgery (Table 3). Mothers’ eating behavior pre-surgery correlated with their children’s eating behavior pre-surgery (UE: *r* = 0.34, *p* = 0.03, and EE: *r* = 0.28, *p* = 0.08), but not at follow-up measurements 9 months post-surgery (UE: *r* = 0.08, *p* = 0.61, and EE: *r* = 0.11, *p* = 0.48) or 4 years post-surgery (UE: *r* = − 0.21, *p* = 0.18, and EE: *r* = − 0.25, *p* = 0.11). Mothers’ BMI pre-surgery or 4 years post-surgery did not correlate with their children’s BMI 4 years post-surgery (*r* = − 0.27, *p* = 0.08 pre-surgery, and *r* = 0.01, *p* = 0.93 4 years post-surgery). Graphs 1–3 (supplementary material) show every child’s individual change in self-concept, body-esteem, and eating behavior over the three time points (pre-surgery, 9 months, and 4 years post-surgery). As seen in supplementary material, the individual differences across the three time points were substantial.

**Table 3:** Children’s self-concept, body-esteem, and eating attitudes, 3 months before and 9 and 48 months after Roux-en-Y Gastric Bypass surgery. Differences within children tested with pairwise *t* test and McNemar’s test

Variable children	Participated/recruited	Pre-surgery, 1st	9 months post-surgery, 2nd	4 years post-surgery, 3rd	1st compared to 2nd	2nd compared to 3rd	1st compared to 3rd
BYI-S^a^ score	37/81	50.5 (6.0)	49.2 (7.6)	46.0 (9.0)	− 1.3 (*p* = 0.268)	− 3.2 (*p* = 0.022)	− 4.4 (*p* = 0.003)
BYI-S percent	37/81	77.8 (17.0)	74.8 (22.2)	65.2 (25.9)	− 3.0 (*p* = 0.353)	− 9.5 (*p* = 0.016)	− 12.5 (*p* = 0.003)
BES^b^ total score	27/81	63.3 (17.7)	61.4 (16.6)	55.9 (19.2)	− 1.9 (*p* = 0.474)	− 5.5 (*p* = 0.099)	− 7.4 (*p* = 0.061)
BES appearance score	33/81	35.1 (10.9)	34.1 (9.7)	31.0 (11.4)	− 1.0 (*p* = 0.500)	− 3.1 (*p* = 0.094)	− 4.2 (*p* = 0.039)
BES weight score	37/81	18.2 (5.9)	17 (5.7)	14.6 (5.9)	− 1.2 (*p* = 0.098)	− 2.4 (*p* = 0.016)	− 3.6 (*p* = 0.001)
BES attribution score	32/81	8.3 (2.4)	8.1 (2.6)	7.1 (2.3)	− 0.2 (*p* = 0.530)	− 1.0 (*p* = 0.055)	− 1.2 (*p* = 0.031)
CHEAT^c^ score	40/81	5.3 (4.0)	4.0 (2.7)	6.0 (4.4)	− 1.3 (*p* = 0.017)	2.0 (*p* = 0.012)	0.7 (*p* = 0.370)

^a^Beck self-concept inventory

^b^The Body-Esteem Scale

^c^The Children’s Eating Attitude Test. Data presented as means (standard deviation)

### Discussion

The main findings of this study were that children of mothers who undergo RYGB had a lower prevalence of overweight and obesity at 9 months, but not at 4 years, after maternal surgery. A similar rebound pattern was observed in terms of improved eating attitudes at 9 months among children, but tendency to rebound 4 years after maternal RYGB. For the mothers, this rebound pattern emerged for sleep quality and symptoms of depression and anxiety. Children’s self-concept and body-esteem decreased over time, without rebound.

### Children’s Weight Status and Eating Behavior

Children’s prevalence of overweight decreased 4 years after their mothers’ RYGB while obesity increased, although none of these results reached statistical significance. Altogether, the prevalence of obesity and overweight was shown to decrease 9 months post-surgery and then increase again 4 years post-surgery. Although these changes lacked statistical significance, earlier published data from the same cohort (when more children were still included) showed a significantly decreased relative risk of overweight and obesity 9 months post-surgery [5]. As expected, the prevalence of overweight/obesity among the children in the current sample was much higher than that seen in Swedish national data from 2011. In our sample, the prevalence of overweight was 58% pre-surgery, compared to around 16% for boys and 13% for girls at the age of 12 in the general population 2011 [25]. Prevalence of overweight/obesity in our sample was comparable with another study of bariatric patients’ children pre-surgery (50% overweight or obese, *n* = 15), although their prevalence of only obesity was much higher than in our sample (37.5% compared to 11.6% pre-surgery) [26]. In Stockholm county, there was no major difference in overweight and obesity prevalence between 8-year-old and 12-year-old children; thus, the increase in obesity seen in our sample over the 4 years between the measurements may not be fully explained by increased age in general [25]. A study by Woodard et al. (2011) found a lower-than-expected BMI in children 1 year after their parent’s gastric bypass surgery. The current study also found a decrease in overweight/obesity among the children after 9 months, although, as in the Woodard et al. study, this was not statistically significant. This may be due to small sample sizes, both in our study and in that by Woodard et al. (*n* = 15) [4]. Additionally, we saw no correlation between mothers’ and children’s BMI trajectories.

In our sample, children improved their eating attitudes (ChEAT scores) from pre-surgery to 9 months post-surgery, but then reported decreased scores again 4 years post-surgery. There are few studies to compare these data with. Woodard et al. found an increase in dieting behavior among 15 children 1 year after a parent’s RYGB surgery [4]. This is not in agreement with our improved ChEAT scores 9 months post-surgery, but it might be difficult to compare these measurements since our questionnaire is more extensive than the single dieting question used in the Woodard et al. study. However, some children improved and some children worsened their ChEAT score in our sample, even though the average was improved 9 months post-surgery. Watowicz et al. compared 45 children with obesity whose parents underwent bariatric surgery to 90 control children, also with obesity, whose parents with obesity did not undergo surgery. Mean follow-up time after parental bariatric surgery was 3.8 years, and mean age of the children was 12.8 years. The study found that children of bariatric surgery patients reported that they more often ate at the “wrong time of the day” and more often had two or more helpings of food compared to control children [27]. It is worth noting that children in a Swedish sample (*n* = 197), specifically girls, had a higher ChEAT score in 8th grade (mean age 14.4 years) compared to 5th grade (mean age 11.4 years), 5.82 among girls and 2.75 among boys compared to 2.03 among girls and 2.55 among boys, respectively [20]. The initial improvement in ChEAT score seen in this study, 9 months post-surgery, might still be present at 4 years post-surgery, and the increase in ChEAT score over the 4 years of this study could be a result of increased age, although we have no comparison group, and therefore, this must remain speculatory.

The mothers’ eating behavior improved significantly post-surgery compared to pre-surgery, mainly driven by improvements in emotional and uncontrolled eating. Cognitive restraint did not decrease post-surgery, it rather increased (although not statistically significant), which might be explained by the effect of the surgery and the dietary recommendations that are given following bariatric surgery. For example, one of the items measuring cognitive restraint in the TFEQ is “I take small portions deliberately”. This is required behavior after RYGB in order to prevent dumping syndrome (an unpleasant phenomenon believed to be caused by sweet and/or fatty food entering the small intestine too quickly) and other gastrointestinal complications such as nausea and vomiting. Thus, the patients must practice cognitive restraint in order to first reduce and then maintain their weight. The eating behavior among our population is in agreement with previous studies [10, 28]. One previous study, by Engström et al. (2015), divided their sample into two groups, good eating control and poor eating control, according to TFEQ-uncontrolled eating scores 2 years post-RYBG [29]. They showed the same pattern as the current study, with a decrease in UE score after 1 year, followed by an increase.

Genetics is also involved in obesity and it has been seen that some epigenetic markers differ in individuals suffering from obesity compared to normal weight individuals [30, 31]. Moreover, it has also been seen that some of these epigenetic markers can alter post-RYGB surgery [30, 31] and additionally they could differ from children born before compared to after maternal RYGB [32]. Berglind et al. found for example differential methylation of genes involved in insulin signaling, T2D, leptin signaling and obesity in siblings born before compared to after maternal RYGB [33]. Even though life style changes and weight loss during pregnancy seems of great importance for children’s epigenetic profile, there might also be a chance for change in epigenetic markers for obesity after birth and therefore there might be a chance to change the higher risk of obesity later in life [32, 34]. RYGB surgery can therefore be of importance not only for the patient performing the surgery but also for their children, especially before they are born. In this study, participating children were already born and had already a higher prevalence of overweight and obesity compared to the general population but it might be possible to change the whole families’ eating habits when the mothers’ eating behavior improved after surgery. Even though parents seem to have an effect on their children’s eating behavior [8] and children’s ChEAT score in our sample followed the same rebound pattern as their mothers’ UE and EE score, we found no correlation between mothers and their children’s eating behavior post-surgery even though they correlated pre-surgery. It seems possible that a mother’s RYGB may be associated with her children’s weight status, but larger samples and the inclusion of a control group would be needed in order to confirm or reject this possibility.

All three dimensions of children’s body-esteem (appearance, weight, and attribution) and self-concept decreased during the 4 years of follow-up and body-esteem were in general lower than a Swedish sample (mean age 14.7 years) [35]. This may be explained by the growing age of the children, since it has been shown earlier among Swedish children and adolescents that body-esteem and self-concept decrease with age [24, 35].

### Mothers’ Changes in Weight, Sleep Quality, and Symptoms of Depression and Anxiety

Contrary to results in the current study, a 2006 meta-analysis by Buchwald showed less weight loss when more than 2 years had passed after RYGB compared to the period before the 2-year follow-up [36]. This was further confirmed in a study by Sjöström et al., whose study showed a weight regain after 4 years [37], even larger than that found by Buchwald et al. However, Sjöström et al. included more RYGB patients than the review from Buchwald, which also included patients who had undergone different bariatric surgery procedures. We did not find a comparable maternal weight regain after 4 years in our sample. This could be due to our small sample size or to differential dropouts, possibly caused by shame, since we visited our participants in their homes. Home visits might be more sensitive for participants who have regained some of their excess weight, leading to higher rates of attrition among the participants who have gained weight between the second and third data collection visits, compared to those who did not gain weight between the two time points.

Karlsson et al. also used HADS to longitudinally (over 10 years) investigate symptoms of depression and anxiety after bariatric surgery, and in agreement with our results, found a large reduction in symptoms of depression after 1 year followed by a rebound [3] to approximately the same level as seen in our study at 4 years post-surgery. In the present study, symptoms of anxiety improved substantially 1 year post-surgery, but similarly to the symptoms of depression, they deteriorated 4 years after surgery, although not to a statistically significant degree. This finding is also in line with those found by Karlsson et al. [3]. The non-significant results seen for the anxiety score might be due to power issues. Scores for both the anxiety and depression subscales were higher before surgery (anxiety: M = 7.1, SD = 5.2; depression: M = 5.1, SD = 3.2) than those of a Swedish general population sample (anxiety: M = 4.8, SD = 3.8; depression: M = 3.8, SD = 3.4) [38], but then improved to become lower than the general population scores 9 months after surgery (anxiety: M = 4.0, SD = 4.4; depression: M = 1.7, D = 1.8), although still within one SD of the normal population. The first year following RYGB, surgery is often described as a “honey-moon period” compared to later time points, with most patients losing weight rapidly and experiencing positive feedback from their surroundings. After this period, weight loss starts to slow down, some may start to regain weight, and this may lead to a reduction in the positive psychosocial changes seen in the period immediately following surgery. It has been speculated that increased depression and decreased health-related quality of life some years after bariatric surgery might be due to weight regain [3, 39], but this suggestion was not supported by the results seen in our sample, since they did not, on average, experience any significant weight regain.

Earlier studies have shown that bariatric surgery may have an influence on patients’ relationship status [40], and the divorce rate after surgery has been suggested to be higher than that of a standard population [41]. In our sample, over 30% of the mothers did not live with the same partner 4 years post-surgery compared to pre-surgery. These results were in line with a previous study with a mean follow-up time of 7.7 years, where 70% of the participants had the same partner at follow-up as before bariatric surgery [42].

### Strengths and Limitations

This study might be considered unique due to the long 4-year follow-up within the family following maternal RYGB. The repeated measurements within the same individuals allow us to follow the mothers and their children longitudinally regarding multiple psychometric instruments across 3 time points. This strength of the participants acting as their own controls is simultaneously the study’s main limitation. With a control group, we would have been able to compare children of mothers with obesity that did not undergo surgery to children of mothers who did undergo RYGB. In practice, it might have been difficult to recruit control participants with obesity and their children to participate in data collection. Furthermore, individuals with obesity who desire RYGB surgery might be different from those who do not, in ways that are unknown and therefore difficult to match or control for. The attrition was quite high at the last data collection time point and might be differential, as discussed above. Some women also chose not to let their children participate in the 4-year follow-up since they had become teenagers and the mothers feared that they might be sensitive about having their weight recorded and being asked about self-esteem, self-concept, and eating behaviors. The children whose mothers expressed these worries might differ from the children who did participate.

## Conclusions

Children of mothers who have undergone RYGB were found to have a lower prevalence of overweight and obesity at 9 months, but not 4 years post-surgery. The same rebound pattern was seen for their eating behavior, and for the mothers’ eating behavior, symptoms of anxiety and depression. This might be related to the mothers’ surgery and could be important for future interventions, to prevent the rebound pattern following RYGB and for decision making whether or not to perform surgery. However, our results must be interpreted with caution, since there was no control group in the current study.

## Electronic supplementary material

ESM 1 (DOCX 135 kb)
