# Supplementary material for: Change in Children’s Self-Concept, Body-Esteem, and Eating Attitudes Before and 4 Years After Maternal RYGB
Source: Obes Surg. 2018 Jun 17;28(10):3276–83. doi: 10.1007/s11695-018-3348-z (PMC6153582; doi:10.1007/s11695-018-3348-z)
Supplement: Supplementary file 1 — (DOCX 135 kb) [file 11695_2018_3348_MOESM1_ESM.docx]

Supplementary materal, article 1: Children’s change in self-concept, body-esteem and eating attitudes before maternal RYGB, and 9 months and 4 years post-surgery, and mothers eating behavior, depression, anxiety and sleep quality.


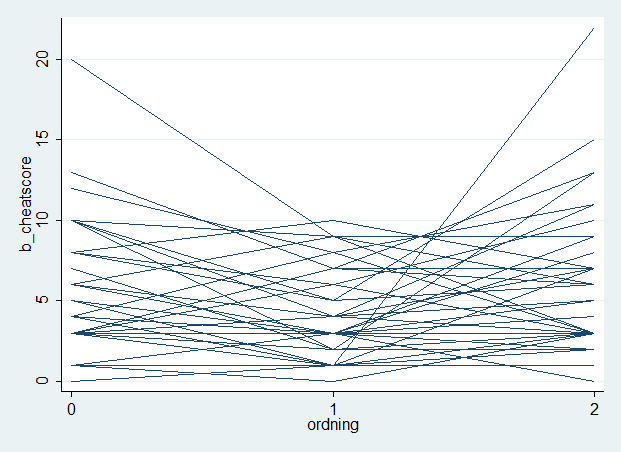

Graph 1, each child’s ChEAT score for each measurement point


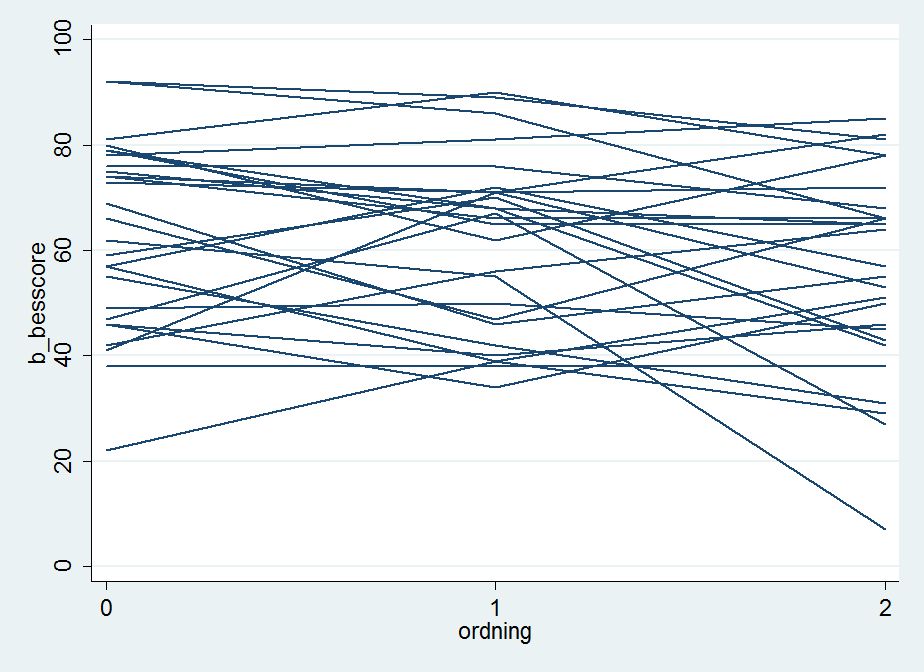

Graph 2, each child’s BES score for each measurement point


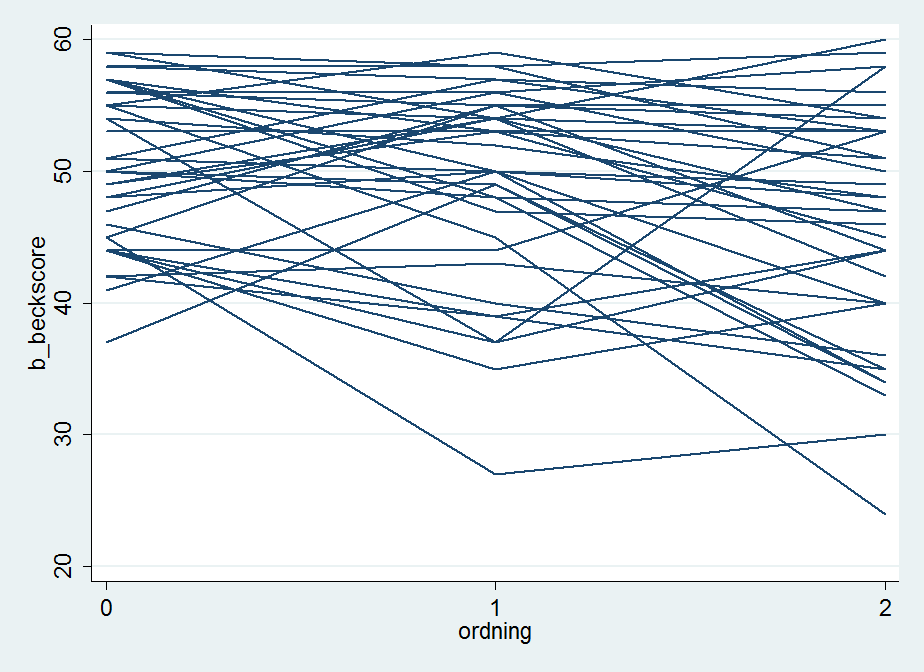

Graph 3, each child’s BYI-S score for each measurement point
